# Predicting self-exclusion among online gamblers: An empirical real-world study

**DOI:** 10.1007/s10899-022-10149-z

**Published:** 2022-08-10

**Authors:** Niklas Hopfgartner, Michael Auer, Mark D. Griffiths, Denis Helic

**Affiliations:** 1 Institute of Interactive Systems and Data Science, Graz University of Technology, Inffeldgasse 16C, 8010 Graz, Austria; 2 neccton GmbH, Davidgasse 5, 7052 Müllendorf, Austria; 3 International Gaming Research Unit, Psychology Department, Nottingham Trent University, 50 Shakespeare Street, NG1 4FQ Nottingham, UK

**Keywords:** Gambling, Responsible gambling, Responsible gambling tools, Problem gambling, Self-exclusion, Voluntary play break, Machine learning

## Abstract

Protecting gamblers from problematic gambling behavior is a major concern for clinicians, researchers, and gambling regulators. Most gambling operators offer a range of so-called responsible gambling tools to help players better understand and control their gambling behavior. One such tool is voluntary self-exclusion, which allows players to block themselves from gambling for a self-selected period. Using player tracking data from three online gambling platforms operating across six countries, this study empirically investigated the factors that led players to self-exclude. Specifically, the study tested (i) which behavioral features led to future self-exclusion, and (ii) whether monetary gambling intensity features (i.e., amount of stakes, losses, and deposits) additionally improved the prediction. A total of 25,720 online gamblers (13% female; mean age = 39.9 years) were analyzed, of whom 414 (1.61%) had a future self-exclusion. Results showed that higher odds of future self-exclusion across countries was associated with a (i) higher number of previous voluntary limit changes and self-exclusions, (ii) higher number of different payment methods for deposits, (iii) higher average number of deposits per session, and (iv) higher number of different types of games played. In five out of six countries, none of the monetary gambling intensity features appeared to affect the odds of future self-exclusion given the inclusion of the aforementioned behavioral variables. Finally, the study examined whether the identified behavioral variables could be used by machine learning algorithms to predict future self-exclusions and generalize to gambling populations of other countries and operators. Overall, machine learning algorithms were able to generalize to other countries in predicting future self-exclusions.

## Introduction

Due to the increasing expansion of the international gambling market (Castrén et al., 2018; Salonen et al., 2018), and the growing use of online gambling (e.g., Chóliz et al., 2021), problem gambling has become an issue of increasing concern. For example, problem gambling frequently leads to negative consequences, such as financial problems or psychological distress for the affected players and their families (Díaz & Pérez, 2021; Dowling et al., 2016). Consequently, many online gambling operators provide responsible gambling (RG) tools to help players prevent overspending and minimize harm from gambling (Harris & Griffiths, 2017), and therefore, RG tools are becoming increasingly common on online gambling platforms (Bonello & Griffiths 2017; Catania & Griffiths, 2021a; Gainsbury, 2014; Lucar et al., 2014; Marionneau & Järvinen-Tassopoulos, 2017). One common RG tool is limit-setting, which allows players to limit the time and money spent over different time periods (typically daily, weekly and/or monthly) (Auer & Griffiths, 2013; Broda et al., 2008; Delfabbro & King, 2021; Ladouceur et al., 2012; Nelson et al., 2008). Feedback on the time and money players spend gambling is another type of RG tool typically offered on numerous online gambling sites (Auer & Griffiths, 2015, 2016a, b; Auer et al., 2014).

Frequently, gambling operators also provide online self-tests in a separate section of the gambling website. Self-tests allow players to reflect on their gambling and its consequences (Jonsson et al., 2017). Furthermore, players are typically also able to exclude themselves from an online gambling site for a limited period or even indefinitely (Caillon et al., 2019; Dragičevic et al., 2011; Griffiths et al., 2009; Motka et al., 2018). Some self-exclusions (also known as voluntary play-breaks) can also be game-type specific, meaning that players only exclude themselves from a specific type of product (e.g., slots, table games, poker, etc.).

According to several studies, the utilization rate of self-exclusion among gamblers is between 5.4% and 11.0% (Jonsson, 2008; Hing et al., 2015; Gainsbury et al., 2020). Furthermore, such time-out tools were unsurprisingly more common among problem gamblers compared to gamblers with low-risk or non-risk gambling. Luquiens et al. (2019) evaluated the effects of self-exclusion in a sample of 4,451 players from an online-poker website. Overall, they found a significant reduction in money and time spent over 12 months after the self-exclusion ended. Players who were the most heavily involved in terms of gambling time also had a significant decrease in time spent gambling after self-exclusion. They therefore concluded that self-exclusion appeared to have positive long-term effects. Previous research has reported that self-exclusion is associated with younger age, higher levels of problem gambling, as well as higher financial losses (Dragičevic et al., 2015; Håkansson & Henzel, 2020). Using a sample of 259 Austrian online players who self-excluded from an online gambling platform, Hayer and Meyer (2011) found that a significant proportion of these players gambled excessively. Based on follow-up interviews, they also suggested that the temporary restriction of access to one single online gambling site had favorable psychosocial effects, because the percentage of potential problem gamblers decreased and there appeared to be almost no switching to other gambling sites.

Several studies have used self-exclusion among online gamblers as a proxy measure for problem gambling (e.g., Finkenwirth, 2021; Haeusler 2016; Percy et al., 2016). Finkenwirth et al. (2021) compared behavioral tracking data from 2,157 self-excluded gamblers against 17,526 players who did not self-exclude. They showed that it is possible to use machine learning models to predict voluntary self-exclusion and highlighted the importance of behavioral data reflecting variability in gambling behavior. Percy et al. (2016) predicted self-exclusion with a sample of 845 online players. They found that the Random Forest machine learning method (Breiman, 2001) scored the highest prediction accuracy of future self-exclusion from online gambling. Haeusler (2016) used transactional data from 2,696 *bwin.com* players to predict future self-exclusion. The frequency and amount of deposits, the variance of withdrawals, the amount of funds subject to reversed withdrawals, and the usage of mobile phone billing were found to be positively associated with self-exclusion. Ukhov et al. (2020) analyzed the gambling behavior of 10,000 online gamblers in the context of problem gambling related self-exclusion. They found that number of cash wagers per active day contributed the most to problem gambling-related exclusion in the case of sports betting, whereas the volume of money spent contributed the most to problem gambling-related exclusion in the case of online casino players.

In the aforementioned studies, self-exclusion was used as a proxy for problem gambling. However, this assumption is controversial. Several studies have shown that problem gamblers may not self-exclude, while non-problem gamblers may self-exclude for reasons other than problem gambling (Griffiths & Auer 2016b; Catania & Griffiths, 2021b; PricewaterhouseCoopers & Responsible Gaming Council of Canada, 2017). On the other hand, there is also literature that has reported relationships between behavior patterns and self-reported/diagnosed problem gambling using survey and interview data (Bagby et al., 2007; McCormack et al., 2013; Sproston et al., 2000). The aim of the present study was to use self-exclusion as a way to identify a set of behavioral markers that were associated with future self-exclusion, and that have also been associated with problem gambling in previous literature. With such markers, operators could target potential high-risk gamblers and introduce them to self-exclusion as an RG tool as early as possible through personalized messaging, as suggested by Motka et al. (2018).

Most previous player tracking studies in gambling research are based on data from one operator and/or one country and depend on monetary gambling intensity features (e.g., amount of money bet, lost, withdrawn, and deposited) to predict RG behavior. However, such models may suffer in generalizing to other countries and operators because online gambling regulations vary significantly across the world. For example, Norway has maximum loss limits and Sweden introduced a maximum deposit limit in June 2020 along with the COVID-19 pandemic. Germany’s new legislation, which was introduced in July 2021, foresees a maximum monthly deposit limit. Moreover, income levels vary between countries and individuals. Therefore, it is important to make no distinction between a high-income player who can afford to bet $10,000 across 1,000 bets and a low-income player who can only afford to bet $100 across 1,000 bets. For that reason, it is important to understand whether monetary gambling intensity features are needed to predict RG behavior because those features may impair the generalizability of machine learning models across countries, operators, and individuals with different income levels. However, the present authors hypothesized that it is possible to understand RG-related behavior, in particular self-exclusions, without considering the actual monetary gambling intensity. If the results support the hypothesis, more general machine learning models could be derived and implemented independently of the jurisdiction or general framework of an operator. Consequently, to address those limitations the present study was designed to answer the following research questions (RQs): (i) What type of behavior correlates with future self-exclusions? (RQ1); (ii) How does monetary gambling intensity affect future self-exclusions? (RQ2); and (iii) Do the identified behavioral variables enable machine learning models to predict future self-exclusions across countries and operators? (RQ3). It is envisaged that the answers to these questions are likely to provide actionable insights for policymakers, and operators who are using algorithm-based RG-tools to prevent problematic gambling behavior.

## Method

The authors were given access to anonymized secondary datasets from three European online gambling operators who provide online gambling in six different countries (i.e., Austria, Germany, Spain, Poland, Sweden, and Slovenia). The raw data contained every game played, every deposit and withdrawal, as well as every self-exclusion and voluntary limit-setting event. Self-exclusions offer the possibility for players to exclude themselves from the gambling website for a self-selected period ranging from 24 hours up to an unlimited time. The data ranged between November 1, 2020 and January 31, 2021.

### Study design

To assess which type of behavior correlates with future self-exclusions, the authors used the player tracking data of November and December to predict whether a player self-excluded in January 2021. The authors only included individuals who had gambled at least on three days in December to have a reasonable amount of player tracking data. This specific threshold represented the median number of active days for all gamblers in the initial dataset. In other words, 50% of all players gambled on at least three days in December. Variations to this threshold with respect to the minimum number of active gambling days in December (i.e., starting from at least one active day up to four active days) yielded statistically but not practically relevant differences in the results and interpretations. Given each individual’s last gambling day in December, the gambling behavior of the previous 30 days prior to the last gambling day in December, was aggregated. Therefore, the resulting number of days until a self-exclusion could occur ranged from one day (i.e., last playing day on December 31, self-exclusion on January 1) to 59 days (i.e., last playing day on December 3, self-exclusion on January 31). This setup enables a reasonable period for a gambling operator to interact with potential future self-excluders and allows a wide range of time periods until a self-exclusion may occur. In summary, the authors utilized past player-tracking data to evaluate which types of behavior were predictive of future self-exclusions.

### Participants

Table 1 reports the number of gamblers, proportion of past and future self-exclusions, average age, and proportion of female gamblers for each country of the three operators after the aforementioned pre-processing steps. In total there were 25,720 gamblers and 414 (1.61%) future self-exclusions. Most of the gamblers in the dataset were from Germany (42.1%) and Austria (29.4%) with a self-exclusion rate of 1.1% and 1.9%, respectively. There was a significant difference in the self-exclusion rate between the countries (chi-square test: χ^2^ = 153.3, *df* = 5, *p* < .001) with Table 2 providing the result of the pairwise comparisons. The mean age of gamblers was 39.9 years, and 13% were female. Both age and the proportion of female gamblers varied significantly between countries (Kruskal-Wallis test: *H* = 1481.9, *df* = 5, *p* < .001, and chi-square test: χ^2^ = 856.2, *df* = 5, *p* < .001 respectively).

**Table 1:** Descriptive statistics of the combined data from the three European online gambling operators.

Country	Number of players	Players with self-exclusions in last 30 days	Players with future self-exclusions	Average age	Female gamblers
Austria	7,526 (29.4%)	14 (0.19%)	141 (1.86%)	39.0	14.3%
Germany	10,822 (42.1%)	159 (1.47%)	118 (1.09%)	42.8	9.6%
Spain	787 (03.1%)	5 (0.64%)	49 (6.23%)	34.3	22.6%
Poland	4,140 (16.1%)	8 (0.19%)	46 (1.11%)	35.3	12.9%
Sweden	877 (03.4%)	17 (1.94%)	30 (3.42%)	45.1	42.1%
Slovenia	1,532 (06.0%)	1 (0.07%)	30 (1.96%)	36.2	9.8%
	25,720 (100%)	204 (0.79%)	414 (1.61%)	39.9	13.0%

**Table 2:** Post-hoc tests for differences in the countries’ self-exclusion rates. Significant *p*-values highlighted in bold according to the Bonferroni corrected alpha-level of 0.05/15 = 0.0033.

	Austria	Germany	Slovenia	Poland	Spain
Germany	**< 0.001**	-	-	-	-
Slovenia	0.8864	0.0052	-	-	-
Poland	**0.0024**	0.9831	0.0196	-	-
Spain	**< 0.001**	**< 0.001**	**< 0.001**	**< 0.001**	-
Sweden	**0.0030**	**< 0.001**	0.0375	**< 0.001**	0.0101

### Statistical analysis

A hierarchical regression analysis was used to compute the association between gambling behavior and voluntary self-exclusions. The dependent variable was a binary metric indicating whether players opted for a voluntary self-exclusion in January 2021. The authors grouped the independent variables into three categories: controls, behavioral features, and monetary intensity features. Appendix 1 provides the full list of features for each of the three categories.

First, the authors created so-called *control models* by performing six regressions (i.e., one regression per country) using the control variables followed by a stepwise backward elimination. As the number of groups (countries, gambling operators) is small, it is difficult to precisely estimate the variation between the groups and therefore a separate regression model for each country was chosen (i.e., no pooling approach) as opposed to a mixed effects or a multilevel model (i.e., partial pooling approach) as recommended by Gelman and Hill (2006). In the stepwise backward elimination, the authors at each step removed the variable with the highest combined *p*-value (Zaykin, 2011) across the six countries until only variables that were significant in at least one country remained. To answer RQ1, the authors followed the same approach as for the control models and performed six regressions using the control and behavioral variables. The resulting models (Models RQ1) were then compared to the respective control models. Similarly, for RQ2, the monetary intensity features were added (Models RQ2), and the resulting models were compared to the respective models without these features (Models RQ1). To test the significance of each feature category and model, a likelihood ratio chi-square test was used. The results of the model comparisons were further confirmed using Akaike’s Information Criteria (AIC). To reduce and prevent multicollinearity among the variables (James et al., 2013), the authors only included variables with a variance inflation factor smaller than 10. The control variables included the players’ age, gender, and account age (i.e., the time since the player created the account). All independent variables were standardized (i.e., zero mean and standard deviation one) for easier comparison of the relative importance. The coefficients are reported for all models. A negative value indicates a decrease in the odds of the corresponding dependent variable, while a positive value indicates an increase in the odds. Finally, Nagelkerkes *R*^2^ (Nagelkerke, 1991) is reported to measure the goodness of fit for all models.

To test RQ3 (i.e., whether machine learning models for predicting future self-exclusion generalize to other countries and operators), the authors fitted five different models, namely AdaBoost, decision trees, extremely randomized trees (extra-trees), gradient boosting, and random forests on all but one country, which served as the test set. To validate the generalizability of the models to different operators and countries, this process was repeated five more times. Each time, a different country served as test set and the other five countries as the training set. This approach allowed each country to serve as the test set once.

To evaluate the practical use of the predictive models, the authors computed a simple model based on one behavioral variable. This baseline model served as a *best guess* approach, and the authors used the binary indicator of whether players had previous self-exclusions in the past 30 days to predict whether players would self-exclude in the future. The authors chose this baseline because it uses a single variable that is available at prediction time and has three favorable properties. First, it predicts a similar proportion of players who will self-exclude in the future (i.e., 0.79% of players had self-exclusions in the past 30 days, compared to 1.69% of players who had self-exclusions in the future; see Table 1). Second, players with a history of self-exclusions are approximately eight times more likely to self-exclude in the future (12.75%) compared to players without a self-exclusion in the past 30 days (1.52%; see Table 3). Third, it hypothesizes that gamblers who have taken steps to control their gambling behavior in the past will do so in the future.

**Table 3:** Descriptive statistics for players with and without self-exclusion in the last 30 days.

Had self-exclusions in last 30 days	Number of players	Players with future self-exclusions	Average age	Female gamblers
No	25,516 (99.21%)	388 (01.52%)	39.9	13.0%
Yes	204 (00.79%)	26 (12.75%)	40.0	13.7%
	25,720 (100.0%)	414 (01.61%)	39.9	13.0%

A randomized cross-validation search was used to find the optimal hyperparameters for each model. Since all five models were tree-based, a common parameter space was used, with the maximum tree depth ranging from two to ten and the proportion of minimum observations per leaf ranging from 0 to 10%. Furthermore, the parameter space for the learning rate of gradient boosting and AdaBoost ranged between 0 and 0.5. For all other parameters, the default values of scikit-learn were used (Pedregosa et al., 2011). The randomized cross-validation search was repeated hundred times for each model to account for variability in the resulting model performance, and average ROC-AUC (Receiver Operator Characteristic-Area Under the Curve) values were reported. To test whether the overall mean performance of the models varied by operator and country, 95% bootstrap confidence intervals were calculated using 10,000 repetitions.

### Ethics

This study was performed in line with the principles of the Declaration of Helsinki and was approved by the research team’s university ethics committee.

## Results

**Fig. 1:**
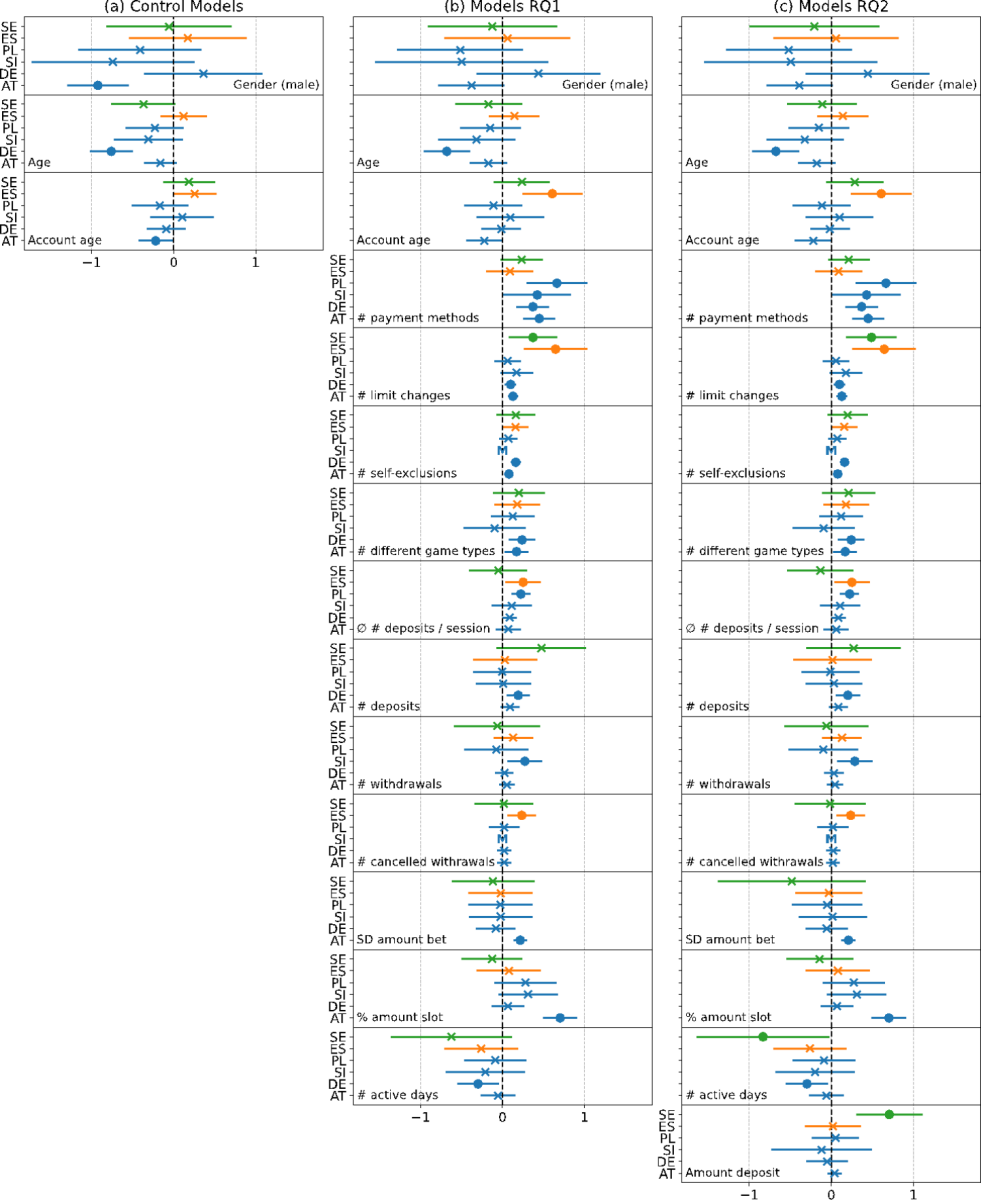
Coefficients including 95% confidence intervals for the hierarchical regression analyses. Each model consisted of six separate regressions (i.e., one for each country) and included only those variables that were significant in at least one country. In Slovenia, there were no previous self-exclusions and cancelled withdrawals, therefore the corresponding coefficient is missing (M) in the figure.

Figure 1 shows the coefficients of the hierarchical regression analyses. The first part of the hierarchical regression analyses (i.e., control models in Fig. 1a) shows the results of six logistic regressions (one per country) with the remaining control variables after backward elimination (i.e., demographics) as predictors. Regarding the demographics of gamblers, the results showed that in Germany, younger gamblers had a significant higher likelihood of self-exclusion. Except for Spain, all other countries also showed a trend toward younger gamblers having a higher likelihood of future self-exclusion. However, these trends were not significant.

The Models RQ1 in Fig. 1b answered the first research question by testing how behavioral features affected future self-exclusions. They showed that a higher number of different payment methods were associated with an increased likelihood of future self-exclusions in the four countries of Operator 1. The coefficients for Spain and Sweden also had a positive sign. However, they were not significant. Frequent limit-setting changes were associated with an increased likelihood of future self-exclusion across operators. Only in Poland and Slovenia were the coefficients non-significant but showed a positive trend. Both previous self-exclusions as well as playing multiple types of games were associated with higher odds of future self-exclusion in Austria and Germany. In the remaining countries, the coefficients also showed a positive trend, except for the coefficient representing the number of different game types in Slovenia.

Overall, the models showed that frequent deposits/withdrawals in general were associated with higher odds of future self-exclusion. More specifically, a higher number of deposits per session was associated with a higher likelihood to self-exclude in Spain and Poland. In Germany, a higher number of deposits in total was associated with higher odds of self-exclusion. Similarly, a higher number of withdrawals was associated with higher odds of self-exclusion in Slovenia, whereas in Spain a higher number of cancelled withdrawals was associated with higher odds in self-exclusions. The models also showed that in Austria a higher percentage of amount bet on slots games compared to the overall bet, as well as a higher standard deviation in the amount bet were associated with an increased likelihood of future self-exclusion. Finally, a lower number of active days was associated with increased odds of self-exclusions in Germany, whereas the coefficients for the remaining countries were not significant but also showed a negative trend. Overall, the inclusion of behavioral features significantly increased the Nagelkerke *R*^2^ (see likelihood ratio chi-square tests in Table 4 for RQ1) and reduced the AIC compared to the model which only contained the control variables. Only in Sweden did the inclusion of behavioral variables not significantly improve the model performance (χ^2^ = 16.3, *df* = 11, *p* = .1304).

**Table 4:** Likelihood ratio chi-square test (LRT), Nagelkerke-*R*^2^ (NK-*R*^2^), and AIC values for the hierarchical regression analyses. Significant *p*-values highlighted in bold according to the Bonferroni corrected alpha-level of 0.05/6 = 0.0083.

		Austria	Germany	Slovenia	Poland	Spain	Sweden
LRT (RQ1)	χ^2^(*df*)	174.6(11)	97.0(11)	24.4(9)	33.4(11)	29.8(11)	16.3(11)
	*p-*value	**< 0.001**	**< 0.001**	**0.004**	**< 0.001**	**0.002**	0.1304
LRT (RQ2)	χ^2^(*df*)	0.54(1)	0.18(1)	0.18(1)	0.10(1)	0.01(1)	10.67(1)
	*p-*value	0.463	0.673	0.667	0.746	0.904	**0.001**
NK-*R*^2^	Models Control	0.025	0.045	0.013	0.012	0.016	0.023
	Models RQ1	0.160	0.124	0.103	0.082	0.115	0.094
	Models RQ2	0.160	0.124	0.104	0.082	0.115	0.139
AIC	Models Control	1377.8	1253.3	299.8	507.7	370.4	264.3
	Models RQ1	1225.2	1178.4	293.4	496.4	362.5	270.0
	Models RQ2	1226.7	1180.2	295.2	498.2	364.5	261.4

The Models RQ2 in Fig. 1c tested the addition of monetary gambling intensity features to the control variables, as well as behavioral features. Except for Sweden, none of the monetary gambling intensity features had a significant additional effect on future self-exclusion. Consequently, the AIC yielded larger values (lower values are generally preferred) compared to the Models RQ1 and the Nagelkerke *R*^2^ stayed at the same level (see likelihood ratio chi-square tests in Table 4 for RQ2). Therefore, given the inclusion of the control and behavioral variables, monetary gambling intensity features appeared to be unrelated to future self-exclusion in five out of six countries.

Finally, a prediction experiment was performed to test whether machine learning models generalize to other countries and operators in predicting future self-exclusions. Table 5 reports the average ROC-AUC scores of hundred training repetitions for all models, gambling operators, and countries. The highest scores were achieved for Operator 1, which operates in four countries. More specifically, the average ROC-AUC value of the best performing models ranged from 0.734 (Poland) to 0.787 (Austria). In Austria, the models achieved a significantly higher average ROC-AUC value than in Germany and Slovenia. There was no significant difference between the average ROC-AUC values of Germany and Slovenia, while the models in Poland achieved a small but significantly lower average ROC-AUC value. Although there were small but significant differences in model performance between some countries, this result suggests that machine learning models can generalize to other countries within a multi-national gambling operator in predicting future self-exclusion.

**Table 5:** Mean ROC-AUC values of the five machine learning models for each country.

Operator	1					2		3
Model	Austria	Germany	Slovenia	Poland		Spain		Sweden
Baseline	0.524	0.548	0.517	0.510		0.518		0.524
AdaBoost	**0.787**	**0.759**	0.755	**0.734**		**0.668**		**0.696**
Decision tree	0.747	0.733	0.724	0.698		0.638		0.678
Extra-trees	0.765	0.724	0.736	0.693		0.579		0.649
Gradient boosting	0.782	0.755	**0.765**	0.721		0.636		0.705
Random forest	0.787	0.757	0.758	0.719		0.606		0.695
Overall mean	0.773	0.746	0.748	0.713		0.625		0.684
95% CI	[0.771 - 0.776]	[0.744– 0.747]	[0.745 - 0.750]	[0.711– 0.715]		[0.622– 0.629]		[0.682– 0.687]

For the single-national Operators 2 and 3, the best performing models achieved an average score of 0.668 (Spain) and 0.696 (Sweden). The average ROC-AUC values were significantly lower for Operator 2 and 3 compared to Operator 1. However, the average ROC-AUC values were significantly higher for Operator 3 than for Operator 2 (see 95% CI in Table 5). Overall, all models outperformed the respective baseline models, which highlights the strength of machine learning algorithms compared to simple rule-based approaches in predicting future self-exclusion behavior.

## Discussion

The present study examined voluntary self-exclusions from three operators with players from six different European countries. There was a large difference with respect to the percentage of self-excluders between the six different countries. Out of the 25,720 active players in December 2020, 414 (1.61%) gamblers self-excluded in January 2021. The largest percentages of self-excluders were found in Spain and Sweden. Both countries have licensed online gambling as well as a national self-exclusion scheme at the time of data collection. In Håkansson and Henzel  (2020) study, 4% of Swedish participants in a web panel reported to have self-excluded. This is similar to the reported percentage of Swedish self-excluders in the present study (i.e., 3.42%).

Results also suggested a tendency for younger gamblers having significantly higher odds of self-exclusion. Håkansson and Henzel (2020) also found a negative association between age and the propensity to self-exclude. Dragičevic et al. (2015) and Haeusler (2016) also reported younger players to be more likely to self-exclude.

The present study computed as many metrics of gambling behavior as possible to reflect wagering, payments, and in-session behavior. The explanatory variables were grouped into (i) control variables (demographics), (ii) behavioral features, and (iii) monetary intensity features. Adding the behavioral features to the control variables significantly increased the explanatory power measured via the Nagelkerke *R*^2^ for all countries except Sweden. The significant behavioral variables in predicting future self-exclusion were the higher number of previous voluntary limit-setting changes and self-exclusions, higher number of different payment methods for deposits, higher percentage of money wagered on slots games, higher average number of deposits per session and in total, higher number of cancelled and total withdrawals, higher standard deviation in amount bet, lower number of active days, and higher number of different game types played.

### Behavioral variables explaining future self-exclusions

The aim of the present study was to identify a set of behavioral variables across countries that might cause gamblers to self-exclude in the future, and which have also been associated with problem gambling in previous literature. Operators could then target potential high-risk gamblers and introduce them to self-exclusion as an RG tool as early as possible through personalized messaging. The behavioral variables that were significantly associated with future self-exclusion across countries (i.e., in at least two countries) are now discussed in relation to problem gambling and what has been found in previous literature.

#### Number of voluntary limit-setting changes

Ivanova et al. (2019) studied the effects of deposit limit prompts on players’ gambling intensity. They found that players who chose to limit themselves without being prompted, spent more money compared to unprompted non-limit setters. They concluded that setting a deposit limit without a prompt or changing a previously set limit may be a marker of gambling problems, which may be used to identify customers in need of help. Therefore, the underlying results of the regression model in the present study support the hypothesis of Ivanova et al. (2019) that changing previously set limits and voluntary use of RG tools (e.g., deposit limits or self-exclusions) may be a marker of gambling problems, as both variables were positively correlated with future self-exclusions, indicating potential gambling problems.

#### Previous self-exclusions

The present study also found that recent self-exclusions were predictive of future self-exclusions. The multiple use of self-exclusions could be an indication of relapse. Hodgins and el-Guebaly (2004) reported that out of 101 pathological gamblers who had recently quit gambling, only 8% were entirely free of gambling during the 12-month follow-up. In an online gambling setting, self-exclusion is a way for gamblers to stop gambling. However, a short self-exclusion time might lead to relapses because players are able to gamble again after a specific time.

#### Deposits per session

The finding of a positive association between the number of deposits per session and the tendency to self-exclude could be an indication of impulsivity. High levels of impulsivity are reported to be associated with problem gambling (Bagby et al., 2007). It is likely that players who show a high level of impulsivity also deposit frequently. Furthermore, Challet-Bouju et al. (2020) analyzed a sample of 1,152 French online lottery players and found that recurrent deposits within a short period (i.e., a high number of deposits per session) were associated with voluntary self-exclusions and higher net losses, which could indicate individuals being potentially at-risk for gambling problems.

#### Number of different payment methods

The number of payment methods used could be an indication of the financial impact gamblers are experiencing. Haeusler (2016) found that self-excluders were more likely to pay for their gambling expenditures by mobile phone billing and less likely to use prepaid cards as a payment method, which highlights the importance of payment methods as an indicator for potentially problematic behavior. Moreover, mobile phone billing as a payment option might be an indication for gambling with money that an individual does not currently have, as the account is usually paid for at the end of the month. Overall, financial problems are one of the most significant impacts of problem gambling (Shaw et al., 2007), therefore increased numbers of payment methods would be expected among problem gamblers.

#### Playing multiple game types

There was also a significant positive association between the number of different game types and the likelihood to self-exclude. This finding is also consistent with previous research showing that those who regularly participated in two or more online gambling activities were significantly more likely to be a problem gambler or at-risk gambler than those who did not participate in two or more online activities (McCormack et al., 2013; Sproston et al., 2000).

### Monetary gambling intensity

The present study also tested whether it was possible to predict voluntary self-exclusion without explanatory variables reflecting monetary gambling intensity. Except for Sweden, the addition of monetary intensity features to the countries’ regression models, which included the control and behavioral features did not significantly increase the explanatory power of the models. More specifically, in five of six countries, none of the explanatory variables reflecting the amount of money gambled, amount of money lost, or amount of money deposited were statistically significant when the models contained the behavioral variables. Apart from the results of Sweden, these findings differ from those of Haeusler (2016) and Ukhov et al. (2020), who found that the amount of money deposited, and the volume of money spent were positively associated with self-exclusion. This is an important finding, given that online gambling regulation varies significantly across countries. For example, the German State Treaty on Gambling, which came into effect in July 2021 restricts deposits to €1,000 per month and Sweden introduced a temporary maximum deposit limit of 5,000 SEK in July 2020. However, the present study showed that it is possible to find statistical patterns predicting voluntary self-exclusion without considering the actual monetary intensity of play.

An exception to these results was Sweden. Indeed, it was only in Sweden that results showed the amount deposited was positively associated with future self-exclusion. The aforementioned study of Ukhov et al. (2020) also comprised Swedish gamblers. One explanation for the amount deposited being a significant variable in Sweden could be the maximum deposit limit of 5,000 SEK (approximately 500 USD), as it prevents high-income players from depositing more than that. For example, a few recreational players who do not self-exclude and can afford to deposit 100,000 SEK could render this variable non-significant, as most problem gamblers who exclude themselves do not have that much money. This hypothesis becomes even more evident when looking at the self-exclusion rates of players ranked in the top 0.5% in terms of amount deposited per country. In Sweden, 40% of the players ranking among the top 0.5% self-excluded, while in Germany, Poland, and Slovenia none of the players ranking among the top 0.5% self-excluded. Overall, these key findings may suggest more general ways to identify at-risk players on the entire financial spectrum among different countries to offer them more targeted RG interventions.

### Generalizability of the identified behavioral variables

The significant behavioral variables were categorized into two groups, namely cross-country variables, and country-specific variables. The group of cross-country variables comprised all variables which were significant in at least two countries, whereas the group of country-specific variables comprised all variables which were significant in only one country. Given that the goal of the study was to examine whether the identified variables can be used to predict future self-excluders (i.e., potential at-risk gamblers) on the entire financial spectrum across countries and operators, only the cross-country behavioral variables were used for the prediction experiments. More specifically, the cross-country variables (i.e., the number of different payment methods, the number of limit changes and previous self-exclusions, and the number of different game types) were used to train five different machine learning models on data from five countries, and the sixth country was used to test the models. This procedure was repeated six times, once for every country.

The overall performance of the models within a multi-national operator covering four countries was similar, with Germany and Slovenia achieving the same performance. However, the model performance on the two single-national operators was significantly lower compared to the multi-national operator. One reason for the varying performance could be the different prevalence of self-exclusion between countries and operators (Zech et al., 2018). Overall, the performance range of the models (ROC-AUC: 0.668–0.787) was similar to the one reported in the self-exclusion study by Finkenwirth et al. (2021) (who reported ROC-AUC: 0.65–0.76). Philander (2014) reported a ROC-AUC range from 0.491 to 0.551, with random forests and neural networks being the highest performing techniques in the prediction of voluntary self-exclusion. Therefore, the results of the present study suggest that machine learning models can generalize to other countries in the context of understanding and predicting voluntary self-exclusion by using pure behavioral variables. Overall, these new insights may help curb potentially problematic gambling behavior.

### Ethical implications

The present study also raises some important ethical implications. While understanding what types of behavior are associated with future self-exclusions may contribute to the development of better RG tools, it could also be used (or more accurately, misused) to reinforce problematic behavior. However, corporate social responsibility and commitment to responsible gambling programs increase customer satisfaction (Abarbanel et al., 2018; Auer et al., 2020; Kim et al., 2017) and therefore player loyalty to gambling operators (Auer et al., 2021). Consequently, it is more ethical and sustainable for gaming operators to use the results of the present study responsibly.

### Practical implications

There are several ways in which the results of the present study can be used by gambling operators, regulators, and researchers. The results showed that there were significant differences in self-exclusion rates between countries and operators. Therefore, operators could use such machine learning models to target potential high-risk gamblers and increase their awareness of self-exclusion as an RG tool through personalized messaging. As suggested by Motka et al. (2018), such an approach could increase the potential of self-exclusion as a player protection measure by increasing the acceptance and utilization of self-exclusion through group-specific information that addresses financial issues and facilitates self-exclusion early in a gambler’s career. Future studies could then examine whether such targeted interventions increase self-exclusion rates among operators/countries that have overall low self-exclusion rates.

### Limitations

Although the present study had a large sample size and contained behavioral tracking data of three gambling operators in different countries, it is not without limitations. As aforementioned, potential differences in the advertisement of voluntary self-exclusions as a responsible gambling tool by operators might have influenced the present findings. It cannot be ruled out that gamblers who did not self-exclude on a platform with an overall low self-exclusion rate might have self-excluded on another platform, where the awareness of such a tool is higher and vice versa. Future studies could address this limitation by using a measure which is independent of the operator’s communication and implementation on their sites.

Furthermore, the age and percentage of female gamblers varied substantial across operators and countries, which could have impacted the generalizability of the machine learning models. For example, in Germany and Slovenia, 9.6% and 9.8% of participants were female, respectively. In Sweden, 42.1% of participants were female, which is similar to the percentage of females reported in a study of Swedish online casino players by Auer and Griffiths (2020). The large gender differences could be due to the different types of games offered, as women are more likely to play bingo and/or slot machines, while men are more likely to play poker and/or bet on sports and horseraces (McCormack et al., 2013). This is in line with the findings of the present study because the gambling operator with players in Austria, Germany, Slovenia, and Poland have a focus on sports betting (although online casino products are also offered). The Spanish gambling operator offers sports betting as well as online casino gambling, but with a larger focus on online casino gambling. The Swedish gambling operator’s product portfolio is restricted to online casino gambling. Future studies could incorporate data from additional gambling operators to diversify the range of games offered, and thereby reflect the entire gambling population more accurately.

There were also several behavioral variables that were significantly associated with future self-exclusion in only one country (i.e., country-specific variables). For example, a higher percentage of money bet on slots games and a higher standard deviation of bets were associated with future self-exclusion in Austria only. Previous studies have found a positive association between slot machine gambling and problem gambling (e.g., Parke & Griffiths 2004, 2006). Similarly, high variation in stakes could indicate a tendency to increase bets to compensate for losses (i.e., chasing losses), which has also been related to problem gambling (Xuan & Shaffer, 2009). Also, the total number of deposits, withdrawals, and canceled withdrawals were significantly associated with self-exclusion in only one country. Finding a better way than merely counting deposits and withdrawals to reflect overall payment behavior could be an interesting avenue for future research. Finally, the authors did not have access to players’ self-reported reasons for self-exclusion. Previous research has shown that self-excluders are a heterogenous population (Catania & Griffiths, 2021b).

## Conclusions

The present study was carried out to understand and predict voluntary self-exclusion based on player tracking data across multiple countries and gambling operators. The results support findings from previous studies with a similar predictive power. Frequent use of RG tools (e.g., limit-setting and self-exclusions), frequent deposits within a session, using multiple payment methods, and playing multiple types of games was significantly associated with the propensity for future self-exclusions. Moreover, machine learning models successfully generalized to the gambling population of other countries. The results also suggested that such models can be applied across gambling operators and countries with a reasonable prediction performance.

Consequently, this is the first real-world study to include data from several operators and several countries. This means that machine learning models in responsible gambling could potentially be applied to different operators and different countries using the proposed behavioral indicators. It also enables new gambling operators that lack historical gambling data to use machine learning models to identify potentially problematic gambling behavior. Overall, much more general markers of harm can be derived from the identified behavioral variables as they do not rely on monetary gambling intensity, which varies across countries. This enables operators to target potential high-risk gamblers and increase their awareness of self-exclusion as an RG tool through personalized messaging.

## Appendix

**Table:** Appendix 1: List of variables and their corresponding definitions

Variable	Description
*Control variables*	Demographics and countries
Germany	German online gamblers
Spain	Spanish online gamblers
Poland	Polish online gamblers
Sweden	Swedish online gamblers
Slovenia	Slovenian online gamblers
Gender	Gender of gamblers (defaults to female)
Age	Age of gamblers in years
Account age	Age of the gambling account in days
*Behavioral features*	All behavioral features were calculated for the last 30 days
Number of self-exclusions	Number of self-exclusions
Number of limit changes	Number of limit changes
Number of deposits	Number of deposits
Std. amount deposits	Standard deviation of the amount deposited
Number of withdrawals	Number of withdrawals
Std. amount withdraws	Standard Deviation of the amount withdrawn
Number of cancelled withdrawals	Number of cancelled withdrawals
Number of bets	Number of placed bets
Std. amount bet	Standard deviation of the amount bet
Number of different games played	Number of different games played
Number of different game types	Number of different game types played (e.g., slot, poker, etc.)
Number of payment methods	Number of payment methods used (e.g., credit card, bank transfer, etc.)
Number of active days	Number of days with at least one bet
Percentage amount slot	Proportion of bet gambled on slot games
Number of sessions	Number of sessions
Percentage of sessions at night	Proportion of session between 1am and 5am
Percentage of sessions at weekends	Proportion of session on Saturday / Sunday
Percentage of amount bet weekends	Proportion of bet gambled on Saturday / Sunday
Avg. session length	Average session length in hours
Avg. number of deposits per session	Average number of deposits per session
Percentage withdrawn	Amount withdrawn divided by amount bet
Percentage deposited	Amount deposited divided by amount bet
*Monetary gambling intensity*	All intensity features were calculated for the past 30 days
Amount bet	Amount bet
Amount deposits	Amount deposited
Amount loss	Amount lost
Avg. amount deposit/session	Average amount deposited per session
